# Leptin Reverts Pro-Apoptotic and Antiproliferative Effects of α-Linolenic Acids in BCR-ABL Positive Leukemic Cells: Involvement of PI3K Pathway

**DOI:** 10.1371/journal.pone.0025651

**Published:** 2011-10-03

**Authors:** Aurore Beaulieu, Géraldine Poncin, Zakia Belaid-Choucair, Chantal Humblet, Gordana Bogdanovic, Georges Lognay, Jacques Boniver, Marie-Paule Defresne

**Affiliations:** 1 Department of Cytology, Histology and Pathological Anatomy (Giga-R), University of Liege, Liège, Belgium; 2 CNRS UMR 8147, Université Paris V René Descartes, Hôpital Necker, Paris, France; 3 Institute of Oncology Sremska Kamenica, Sremska Kamenica, Serbia; 4 Department of Analytical Chemistry, University of Liege, Gembloux, Belgium; Florida International University, United States of America

## Abstract

It is suspected that bone marrow (BM) microenvironmental factors may influence the evolution of chronic myeloid leukaemia (CML). In this study, we postulated that adipocytes and lipids could be involved in the progression of CML. To test this hypothesis, adipocytes were co-cultured with two BCR-ABL positive cell lines (PCMDS and K562). T cell (Jurkat) and stroma cell (HS-5) lines were used as controls. In the second set of experiments, leukemic cell lines were treated with stearic, oleic, linoleic or α-linolenic acids in presence or absence of leptin. Survival, proliferation, leptin production, OB-R isoforms (OB-Ra and OB-Rb), phosphoinositide 3-kinase (PI3k) and BCL-2 expression have been tested after 24h, 48h and 72h of treatment. Our results showed that adipocytes induced a decrease of CML proliferation and an increase in lipid accumulation in leukemic cells. In addition, CML cell lines induced adipocytes cell death. Chromatography analysis showed that BM microenvironment cells were full of saturated (SFA) and monounsaturated (MUFA) fatty acids, fatty acids that protect tumor cells against external agents. Stearic acid increased Bcl-2 expression in PCMDS, whereas oleic and linoleic acids had no effects. In contrast, α-linolenic acid decreased the proliferation and the survival of CML cell lines as well as BCL-2 and OB-R expression. The effect of α-linolenic acids seemed to be due to PI3K pathway and Bcl-2 inhibition. Leptin production was detected in the co-culture medium. In the presence of leptin, the effect of α-linolenic acid on proliferation, survival, OB-R and BCl-2 expression was reduced.

## Introduction

Chronic myelogenous leukemia (CML) is a malignant disorder of the hematopoietic stem cell (HSC) characterized by a reciprocal translocation between chromosomes 9 and 22 (t(9;22)(q34;q11)) [1]. The translocation results in formation of the BCR-ABL fusion oncogene encoding a protein with constitutive tyrosine kinase activation, which plays a central role in the pathogenesis of the disease. Many mechanisms are involved in the malignant transformation orchestrated by the BCR-ABL oncoprotein [2]. It constitutively activates mitogenic signaling pathways such as the phosphoinositide-3 (PI3) kinase pathway [3]. Once activated, the PI3K controls cell growth, proliferation and apoptosis, as well as steps that are involved in tumor formation and malignant cell dissemination [4].

Although abnormal leukemic cells define the tumor compartment itself, environmental factors could participate in the evolution of the disease. Indeed, alterations in the bone marrow microenvironment were widely described. These alterations include deregulated patterns of cytokine production that promoted a proinflammatory environment. These alterations induce a deficient of hematopoietic supportive capacity and decreased the number of cells in certain stromal cell populations [5].

In addition: 1) Some reports suggest that obesity play important role in CML risk [6]–[7]. ; 2) Morphologically identifiable adipocytes, the most abundant bone marrow stromal cells in healthy adult [8]–[9], were shown to inhibit myeloid differenciation [10], [11]. In patient with CML, the number of pre-adipocytes increased during the chronic phase and was reduced once CML entered blast phase [12]. At remission, there is a recurrence of bone marrow (BM) adipocytes [13]. ; 3) In certain cases of leukemia, a shift of low density lipoprotein (LDL) from blood to spleen, liver and bone marrow was observed [14]–[15]. At the remission, LDL in peripheral blood return to normal values.A drop in saturated and monounsaturated fatty acids in the bone marrow was also described in leukemic patient [16], [17]. The involvement of fatty acids has been widely demonstrated in some cancers such as breast cancer [18]–[19] but has never been studied in CML. ; 4) Genes coding for the receptor of leptin (OB-R), an adipokine produced predominantly by adipocytes [8], [20], and for proteins involved in fatty acid synthesis, were upregulated in CML cells [3]. This upregulation was directly associated with activation of the PI3K signaling pathway, the major signalling pathway of OB-R [21], involved in drug resistance [22]. ; 5) Despite many studies demonstrating the protective effects of leptin against acute myeloid leukemia (AML) [20], [23], the role of leptin in CML remains unclear. CML seems to be resistant to the proliferative effects of leptin [20].

Lipid accumulation in the BM associated with OB-R expression and PI3K/AKT signalling activation led us to believe that fat cells and lipids could be involved in the evolution of the disease. In this work, we studied the possible role of adipocytes, leptin and fatty acids in the CML disease progression by analysing interactions between BM adipocytes and BCR-ABL positive cell lines and exploring the potential effects of leptin and fatty acids on these cell lines.

## Materials and Methods

### Isolation and culture of adipocytes

Femoral and iliac crest bone marrow (BM) samples were obtained from patients undergoing hip surgery at the Department of Orthopedic Surgery (CHR, Liège, Belgium). The hospital ethics committee of the University of Liège (B70720095686-2009/26) approved this study and the donors were informed by written consent in accordance with the Helsinki convention. Adipocytes were isolated from femoral biopsies and cultured as previously described [10]: briefly, after dissociation with collagenase, floating adipocytes were cultured using the ceiling method. They were amplified in a long-term culture medium (alpha-MEM, 12.5% horse serum, 12.5% fetal calf serum, 2mM L-glutamax, 0.2M inositol, 100 M ß-mercaptoethanol, 50 U/ml penicillin and 50 µg/ml streptomycin). Adipocytes from BM acquired a fibroblast-like fat cell (FLFC) morphology *in vitro* but conserved the typical features of freshly isolated BM adipocytes [10].

### Cell lines

PCMDS cell line, was described as CML [24] and kindly provided by Dr G.Bogdanovic (Sremska Kamenica Oncology Institute, Serbia). A stromal cell line (HS-5), the CML cell line K562 and a T cell line (Jurkat) were purchased from American Type Culture Collection (ATCC Rockville, MD). Cell lines were maintained in RPMI-1640 medium containing 10% fetal calf serum, 1% L-glutamine, 50 U/ml penicillin and 50 µg/ml streptomycin.

### Co-cultures of leukemic cells with FLFCs or HS-5

FLFCs or HS-5 were plated in 6- or 96-well plates at 100×10^3^ or 30×10^3^ cells, respectively. After monolayers formation, 5×10^3^ (96-well plates) or 30×10^3^ (6-well plates) PCMDS, K562 or Jurkat cells were added either in cell-cell contact or using transwell chamber (cell culture insert 8 µm pore size PET track-etched membrane, Becton Dickinson, France). For [^3^H]Thymidine experiments, FLFCs and HS-5 monolayers were irradiated before the addition of 10×10^3^ PCMDS**.** Cultures were stopped after 24h, 48h and one week. Conditioned media were collected and stored at −80°C until dosage of leptin by enzyme-linked immunosorbent assay (ELISA)(R&D Systems Inc, France). Leukemic cells were used for morphological analysis, immunostaining, protein and RNA extraction.

### Fatty acid stimulation

For lipid stimulation, PCMDS, K562 or Jurkat cell line (25×10^3^ /ml) were cultured in the presence of 50 to 250 µM of stearic (C18∶0), oleic (C18∶1), linoleic (C18∶2 n-6) or α-linolenic acids (C18∶3 n-3) (all from Sigma-Aldricht, France) with or without addition of 400 µg/ml of α-Tocopherol (Sigma-Aldricht, France).

### Oil Red O staining

Cytospins were realized for Oil Red O staining : they were fixed in formaldehyde vapor during 5 min, stained with Oil-Red-O (Sigma-Aldricht, France) for 10 min, air-dried and counterstained with hematoxylin for 5 min.

### Flow cytometr**y**

Flow cytometry analysis were performed on a FACS-CantoII (Becton Dickinson and Company, France). Cell suspensions were incubated with a mouse anti-OBR (R&D Systems, France) antibody (1/50) for one hour at room temperature, then with a FITC conjugated rat anti-mouse (IgG2b) antibody (1/400) (BD biosciences, France).

### [^3^H]Thymidine uptake

After 24h or 48h of co-culture, 0.1 µCi of [^3^H]Thymidine (specific activity, 6.7 Ci/mmol), (DuPont, Wilmington, DE) was added to the medium for 6 hours. The cell suspensions were then deposited onto harvester filters using a semiautomatic cell harverster (unifilter 96 haverster, Perkinelmer, USA). Radioactivity was measured using a scintillation counter (Topcount, Perkinelmer, USA) and expressed as counts per minute (CPM).

### Apoptosis

To quantify apoptosis, the cells were analyzed over time by staining for the phosphatidylserine translocation using FITC-annexin V and propidium iodide (PI) (Annexin-V/PI kit, BD Pharmingen, USA), according to the manufacturer's instructions. Samples were analysed on a FACS CantoII (Becton Dickinson, France).

### Fatty acids analysis

Total lipids were extracted from femoral bone marrow with chloroform-methanol 2∶1 (v:v) as previuosly described Folch et al [25]. The extract was concentrated under reduce pressure at 35°C. Fatty acid methyl esters (FAME) were prepared from crude lipids by Boron trifluoride catalyzed transesterification. Ten mg lipids were dissolved in 0.5 ml n-hexane to which 0.5 ml of reagent (14% BF3-methanol, dry methanol and n-hexane 25∶55∶20 v/v/v) were added in 10 ml sovirell tubes. The sealed tubes were maintained at 70°C during 90 min. After cooling 0.1 ml 10% H_2_SO_4_ and 0.5 ml saturated solution of NaCl were added and the solution diluted in 8 ml of pure n-hexane. FAME were analysed by gas liquid chromatography on a Agilent 6890 apparatus fitted with cold “on-column” injection and a FID detector (maintained at 250°C). The operating conditions were as follows: 30 m×0.25 mm, Factor four MAX from Varian, film thickness: 0.25 µm; temperature programme: from 50°C (hold of 1 min) to 150°C at 30°C /min and from 150°C to 230 at 5°C /min with a time final hold of 18 min. Helium at 70 KPa was used as carrier gas. FAME were identified on the basis of their retention time with those of the SUPELCO 37 FAME MIX (47885-U). The results for each FAME are expressed in area %.

### Western immunoblotting

Cell lysates were prepared with the use of RIPA buffer (10 mM Tris [tris(hydroxymethyl)aminomethane], pH 7.4; 150 mM NaCl; 1% Triton ×−100; 0.5% deoxycholate; 0.1% sodium dodecylsulfate [SDS]; 5 mM ethylenediaminetetraacetic acid [EDTA]) containing protease inhibitors (complete tablets; Roche, Basel, Switzerland). Aliquots of protein samples (50 µg) or equivalent amounts of cells (2×10^6^ cells) were mixed with the same volume of double-strength Laemmli buffer (125 mM Tris–HCl pH 6.8, 4% SDS, 20% glycerol, 10% 2–mercaptoethanol, and 0.002% bromophenol blue). The samples were boiled for 5 min and subjected to SDS-polyacrylamide gel electrophoresis (PAGE) (7-12% gradient gels). Immunobloting was performed using monoclonal antibodies or polyclonal antisera against actin (A 2066, Sigma); BCL-2 (DAKO A/S, Glostrup, Denmark); leptin receptor (R&D systems, France); AKT, Phospho-Akt (Ser473), (9272, 9271,respectively; Cell Signalling, Danvers, MA 01923). Immunodetection was performed by the using of horseradish peroxidase-conjugated secondary antibodies (mouse IgG or rabbit IgG, horseradish peroxidase linked whole antibodies, NA931 or NA934, Amersham, Buckinghamshire, UK) and an enhanced chemiluminescence method (RPN2132; Amersham, Buckinghamshire, UK) involving exposure to X-ray film (Amersham hyperfilm ECL, Buckinghamshire, UK).

### RNA extraction and Reverse Transcription-Polymerase Chain Reaction Analysis

Total RNA was purified from cell suspensions using the High Pure RNA Isolation kit (Roche Diagnostics, Germany). The amount of purified RNA was quantified by fluorimetry using the RiboGreenTM RNA Quantification kit (Molecular Probes Eugene, USA). Those specific primers were used to amplify the following mRNA: endogens 28S: 3′-GTTCACCCACTAATAGGGAACGTGA and 5′ GATTCTGACTTAGAGGCGTTCAGT OB-Rb: 5′-GCCAACAACTGTGGTCTCTC and 3′-AGAGAAGCACTTGGTGACTG; OB-Ra: 3′-AAGGAGTGGGAAAACCAAAG and 5′-CCACCATATGTTAACTCTCAG; Reactions were performed in an automated thermal cycler (Perkin-Elmer, USA) using the geneAmp Thermostable rTth Reverse Transcriptase RNA PCR kit (Perkin-Elmer, USA), specific pairs of primers (Eurogentec, Belgium), 10 ng of RNA per 25 µl reaction mixture. Known copy number of the internal standard (ssRNA) was used for 28S mRNA amplification.

### Statistical analysis

Experiments were performed with at least three independent determinations. The results were analyzed by independent samples two-tailed and unpaired Student's t-test, and were presented as means ± standard deviation (SD).

## Results

### Direct contact between FLFCs and PCMDS induced lipid accumulation in PCMDS and inhibited their proliferation

It was previously shown that FLFCs obtained from BM adipocytes exerted a negative control on granulocyte differentiation [10]. This observation raised the question of wether FLFCs influenced leukemic myelomonocytic cells. To address this issue, FLFCs were cultured with a BCR-ABL positive myelomonocytic leukemic cell line, PCMDS and HS-5 stromal cell line was used as control. The co-cultures were realized either in direct contact or in transwell conditions and stopped after 48 hours.

**Figure 1:**
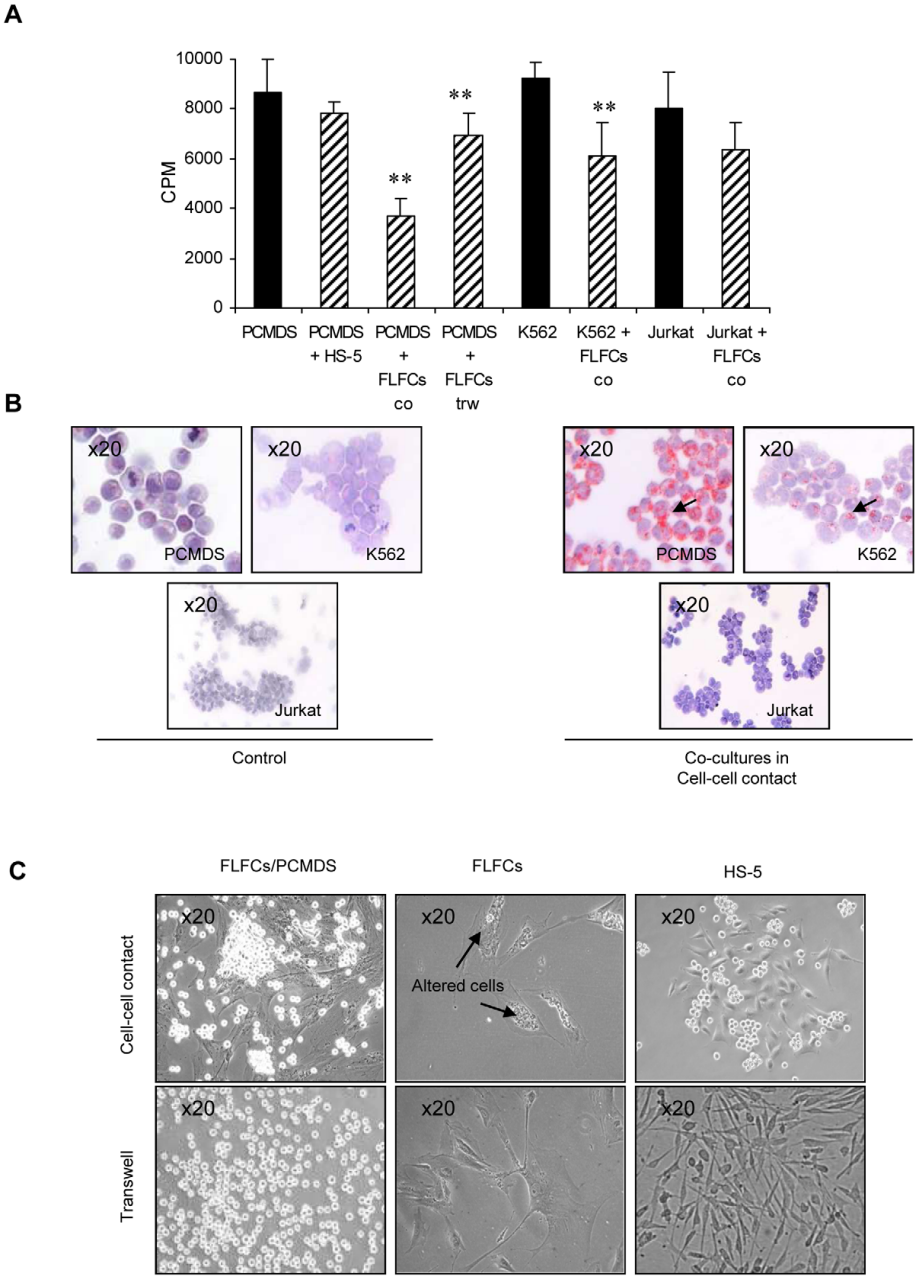
Coculture of FLFCs and PCMDS for 48h: Influence on PCMDS. **a** PCMDS proliferation in direct contact (co) and in transwell (trw) conditions with FLFCs and HS-5 stromal cell lines vs K562 and Jurkat cells in direct contact with FLFCs. **P<0.01 versus control cells (*black columns*) (Student *t* test). *Columns*, mean of at least three independent determinations, *bars*. SD. **b** lipid accumulation was observed by Oil Red O staining in PCMDS and in K562 in direct contact and in transwell condition (not show) but not in Jurkat cells (magnification, x20) → shows intracytoplasmic lipid droplets. **c** cell-cell direct contact with PCMDS induce morphological alterations in FLFCs that are not observed in transwell condition (magnification, x20).

In both conditions, PCMDS proliferation rate as tested by [^3^H] thymidine uptake was significantly (p<0.01) lower (3683.75±733.48 cpm) in the presence of FLFCs than in control conditions (8678.5±1320.92 cpm) or in the presence of HS-5 (7829.5±462.79 cpm). This decrease in proliferation was also detected with another BCR-ABL positive cell line, K562 cells (p<0.01) (921.14±68.04 cpm in control condition vs 614.71±132.19 cpm in co-culture with FLFC) but not with Jurkat cells (p = 0.184450) (805.85±142.73 cpm in control conditions vs 637.42±107.14 cpm in co-culture with FLFCs) (Fig. 1a). Cell survival was similar in all the culture conditions tested (data not shown). Morphological examination after Oil Red O staining showed that PCMDS and K562 cells but not Jurkat cells accumulated lipid droplets in their cytoplasm when co-cultured with FLFCs (Fig. 1b) but not with HS-5 (not shown). Furthermore, when the co-cultures were stopped after one week, morphological examination showed that, in direct cell-cell contact, but not in transwell conditions, FLFCs but not HS-5 displayed morphological alterations suggesting cell death in the presence of PCMDS (Fig. 1c).

### Contact between FLFCs and PCMDS induced leptin production and increased OB-R expression on leukemic cells

**Figure 2:**
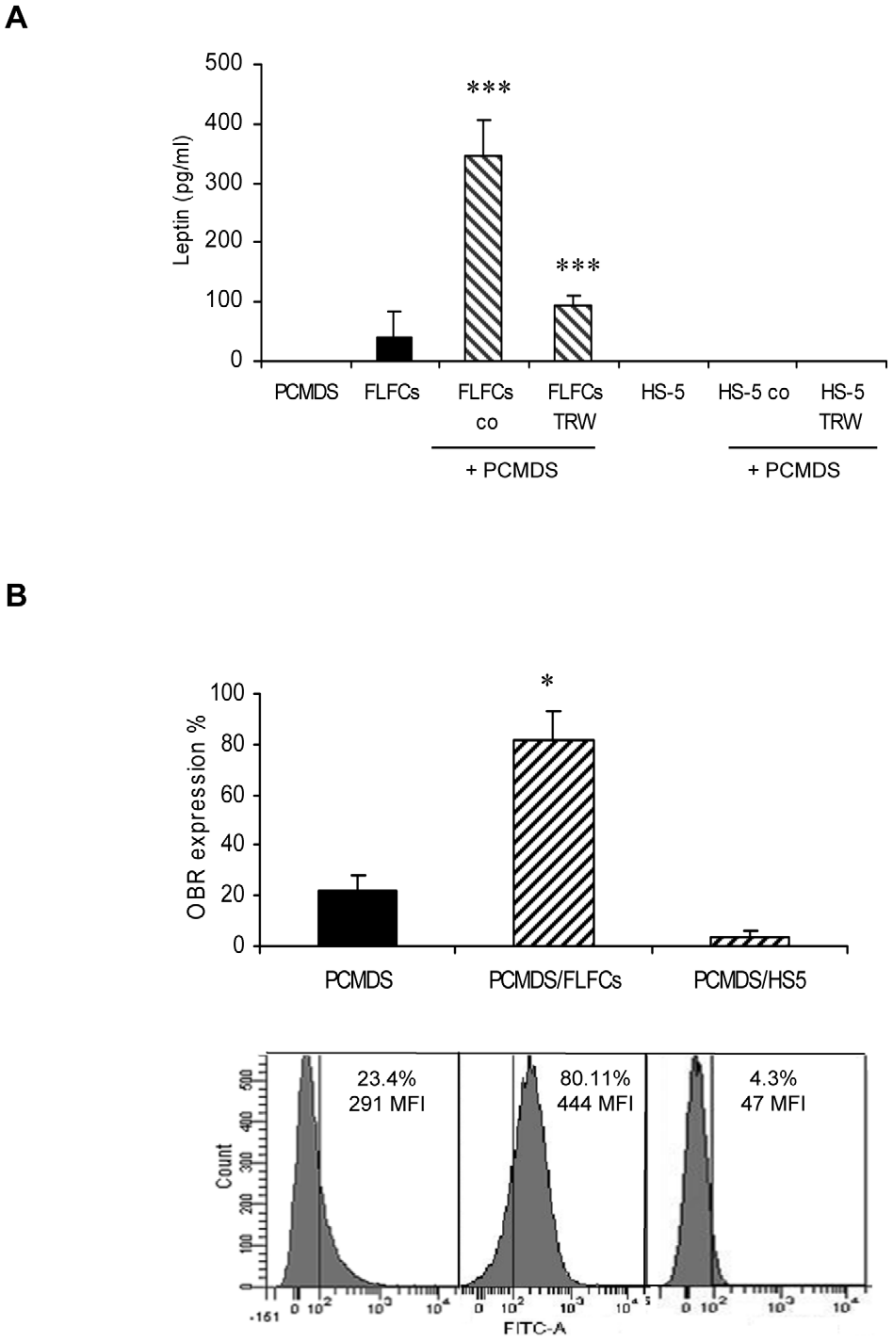
Coculture of FLFCs and PCMDS: Leptin production and OB-R expression. **a** leptin production assessed by enzyme-linked immunosorbent assay in direct contact (co) and transwell (trw) coculture of PCMDS with FLFCs or HS-5 stromal cell lines after one week (*black columns* no contact; *hatched columns* direct end transwell contact). ***P<0.001 versus PCMDS in control condition (Student *t* test). **b** after 48h, leptin receptor (OBR) expression was analysed by flow cytometry after immunostaining of PCMDS cultured in control conditions (*black columns*) or in co-culture with FLFCs or HS-5 (*hatched columns*).*P<0.05 versus PCMDS in control condition (Student *t* test). *Columns*, mean of at least three independent determinations, *bars*. SD.

As adipocytes exert many effects on hematopoietic cells by producing leptin [26], we have compared leptin production by FLFCs and HS-5 and OB-R expression on the cell surface of PCMDS in control (medium alone) and co-culture conditions (Fig 2A and 2b). Leptin levels measured by Elisa in co-culture of FLFCs with PCMDS in direct cell-cell contact were greatly increased (p<0.001), compared to cultures of FLFCs alone (344.68±59.7 pg/ml vs 38.67±43.67 pg/ml) or to co-culture of FLFCs with CD34 progenitors (120 pg/ml) [10]. In transwell conditions, leptin level (92.38±17.26 pg/ml) was also significantly increased (p<0.001) but to a lesser extent than in direct cell-cell contact. No leptin was detected in co-cultures of PCMDS with HS-5.

OB-R expression was tested by flow cytometry. In control condition, 21.63% of PCMDS expressed OB-R with a mean fluorescence intensity of 252±3.1. The percentage of PCMDS expressing OB-R (p<0.05) as well as the fluorescence intensity (p<0.05) greatly increased when they were co-cultured with FLFCs but not with HS-5 (81.86±11.16% and 446.33±2 MFI versus 3.35±2.43% and 44.6 ± 26.71 MFI) (Fig. 2b).

### Lipid composition of normal bone marrow

As we observed lipid accumulation in PCMDS and K562 cytoplasm when they were cultured in contact with FLFCs, we measured the lipidic content in four bone marrow biopsies and two BM aspirates (Table 1). The total extractable lipid in the bone marrow specimens varied between 19.4 to 35.2% of fresh tissue weight. In BM biopsies, saturated fatty acids (SFA) accounted for 41.33±4.6%, monounsaturated fatty acids (MUFA) for 48.38±4.11% and polyunsaturated acids (PUFA) for 5.73±2.516% of the total whereas in BM aspirates, SFA accounted for 36.27±2.78%, MUFA for 36.28±9.41% and PUFA for 12.69±0.5%.

**Table 1:** Fatty acid composition of bone marrow aspirates and biopsies.

Fatty acids	Bone marrow aspirates	Bone marrow biopsy
Saturated fatty acids	36.27±2.78%	41.335±4.6%
Monounsaturated fatty acids	36.28±9.41%	48.38±4.11%
Polyunsaturated fatty acids	12.695±0.5%	5.735±2.51%

Fatty acids was analysed by gas lipid chromatography as described in “Materials and methods”**.** Values expressed as mean ± SD (standard deviation)

### Stearic saturated fatty acids (SFA), oleic monounsaturated acid (MUFA) and linolenic polyunsaturated fatty acids (PUFA) differentially influenced PCMDS proliferation and survival, as well as Bcl-2 and OB-R expression

**Figure 3:**
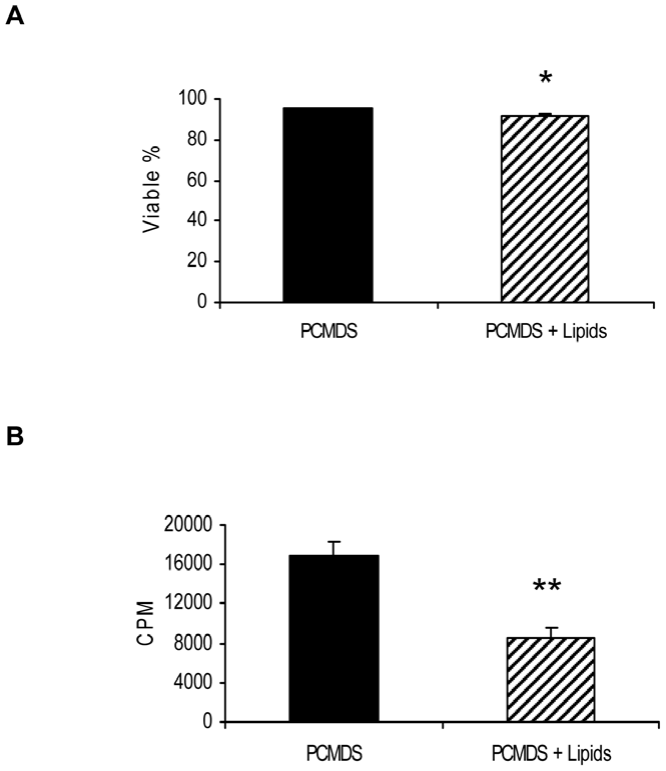
PCMDS in a medium supplemented with lipids mixture for 24h. PCMDS viability (**a**) and proliferation (**b**) decrease in contact with lipids (*black columns* no lipids stimulation; *hatched columns* lipids stimulation). * *P*<0.05 and **P<0.01 versus PCMDS without lipids treatment (Student *t* test). *Columns*, mean of at least three independent determinations, *bars*. SD.

**Figure 4:**
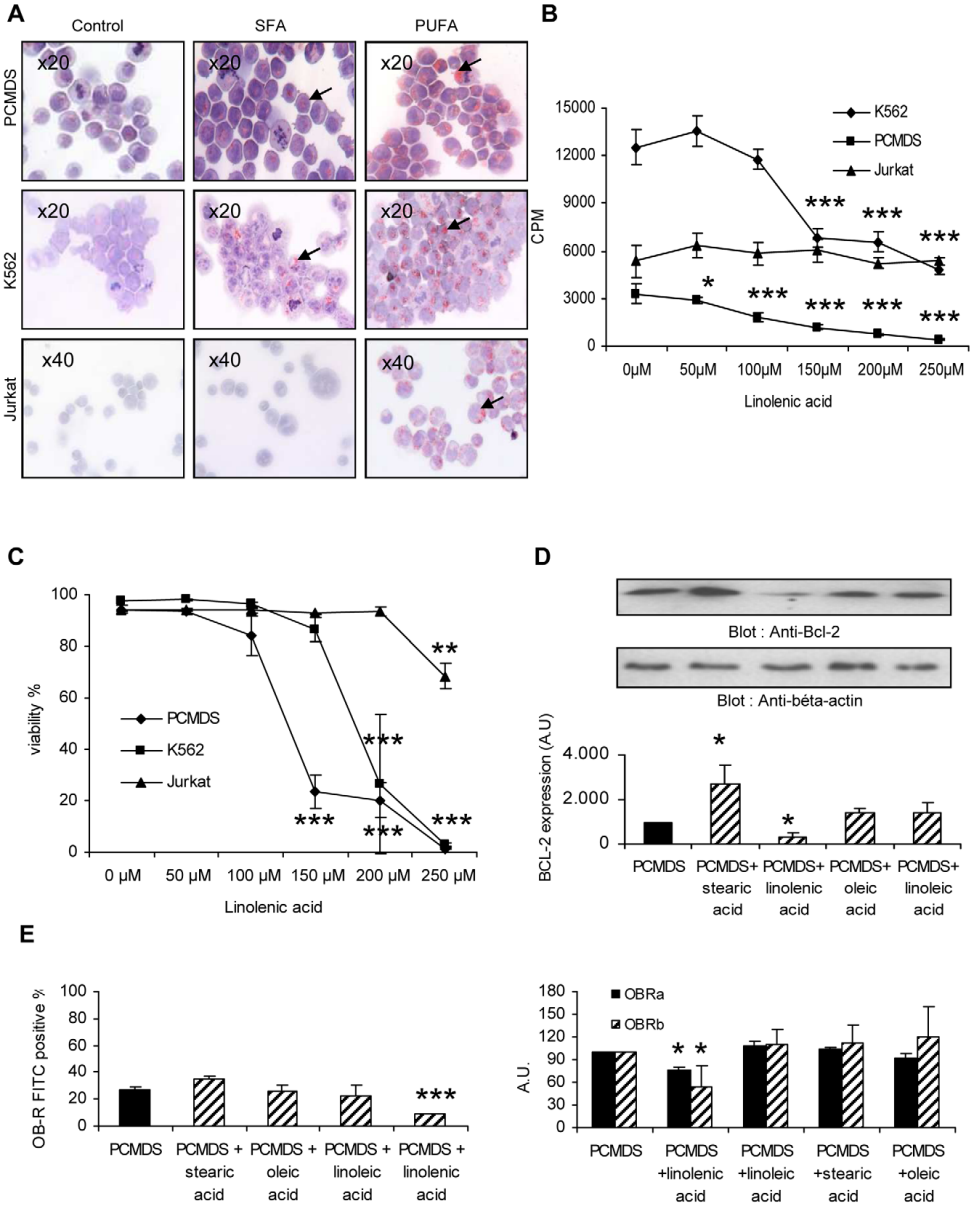
PCMDS, K562 and Jurkat cells cultured with stearic, oleic, linoleic and α-linolenic acids. **a** lipid accumulation in PCMDS, K562 and Jurkat cell in contact with saturated (SFA) and polyunsaturated (PUFA) fatty acids (magnification, x20 and x40) → shows intracytoplasmic lipid droplets. **b** effects of α-linolenic acid on PCMDS, K562 and Jurkat cells proliferation after 48 hours. *P<0.05 and ***P<0.001 versus in control condition (Student *t* test). **c** effects of α-linolenic acid on PCMDS, K562 and Jurkat cells survival after 48 hours. **P<0.01 and ***P<0.001 versus in control condition (Student *t* test). **d** effects of 200 µM of α-linolenic, linoleic, oleic and stearic acids on Bcl-2 expression (*black columns* no lipids stimulation; *hatched columns* lipids stimulation) measured by western blot. *P<0.05 versus PCMDS in control condition (Student *t* test). **e** effects of 200 µM of α-linolenic, linoleic, oleic and stearic acids after 24 hours on OB-R (*black columns)* no lipids stimulation; *hatched columns* lipids stimulation) and on OB-Ra (*black columns*) and OB-Rb (*hatched columns*) mRNA expression in PCMDS. *P<0.05 and ***P<0.001 versus in control condition **(**Student *t* test) *Columns*, mean of at least three independent determinations, *bars*. SD.

The accumulation of lipids in myelomonocytic leukemic cells in the presence of FLFCs led us to investigate wether the effects of FLFCs were at least partially due to the fatty acids they contain. To address this question, PCMDS were cultured in a medium containing approximatively the same proportion (33%) of lipids and the same composition (50% oleic, 43% stearic, 6.5% linoleic and 0.5% α-linolenic acid) than that observed in a bone marrow biopsy. After 48 hours, PCMDS viability and proliferation were significantly decreased (95.13±0.11% viability in control conditions vs 91.75±0.61% in the presence of lipids, p<0.05 and 16826±1537.27 cpm in controls vs 8555.33±1086.23 cpm in the presence of lipids, p<0.01)(Fig.3a and 3b). To assess whether the different types of fatty acids equally affected PCMDS survival and proliferation, we cultured PCMDS, K562 and Jurkat cell lines in presence of 50 to 250 µM of saturated stearic acids (SFA) or of monounsaturated (MUFA) oleic acids or of polyunsaturated (PUFA) linoleic acid and α-linolenic acids. This range of concentration corresponded to that observed *in situ*. Lipid accumulation in their cytoplasm was studied by Oil Red O staining. In the presence of PUFA, PCMDS and K562 accumulated more lipids than in the presence of SFA. This accumulation was also observed in Jurkat cells (Fig.4a). After 48 hours, α-Linolenic acid accumulation was associated with a decrease of PCMDS and K562 proliferation (p<0.001)(100 µM: 1849.28±304.33 cpm, 150 µM: 1189.57±166.70 cpm, 200 µM: 763±42.50 cpm and 250 µM: 394.14±47.04 cpm vs 3301.71±603.50 cpm, in control) (150 µM: 6822.57±587.42 cpm, 200 µM: 6569.85±609.80 cpm and 250 µM: 4792.57±280.26 cpm vs 12535.28±1131.70 cpm, as control) and survival (p<0.001)(150 µM: 23.48±6.41%, 200 µM: 20.2±6.83% and 250 µM: 1.43±0.32% vs 94.31±1.59%, as control)(150 µM: 86.6±4.80%, 200 µM: 26.7±27.01% and 250 µM: 2.85±0.49% vs 97.8±0 as control) in a dose dependant manner (Fig. 4b and 4c). Only high amounts (250 µM) of α-linolenic acids slightly affected Jurkat cell survival (p<0.01) (68.46±5.22% vs 93.93±0.35%) but without any influence on their proliferation (Fig. 4b and 4c). Linoleic (PUFA), oleic (MUFA) and stearic acids (SFA) did not affect neither proliferation nor survival of PCMDS and K562 (results not shown).

Linoleic (PUFA) and oleic acids (MUFA) did not influence Bcl-2 expression whereas, after 24 hours stearic acids (SFA) increased (p<0.05) and α-linolenic acids (PUFA) decreased (p<0.05) its expression in PCMDS (2732±797 A.U. and 508±150 A.U. respectively vs 1000 A.U. in control conditions) (Fig. 4d). The decrease of Bcl-2 expression in presence of α-linolenic acids was correlated to the low level of survival induced by this fatty acid (Fig. 4b).

As BCR-ABL positive chronic myeloid leukemia cells express leptin receptor (OB-R) [3] and as we observed an increase of OB-R expression in PCMDS co-cultured with FLFCs, we evaluated whether fatty acids could affect the expression of OB-R after 24 hours of culture. Our results showed that stearic, oleic and linoleic fatty acids (200 µM) did not modify OB-R expression (34.7±2.42% ; 25.96±3.83% ; 22.08±8.67%, respectively vs 27.16±2.11% in control conditions). On the other hand, 200 µM of α-linolenic acid induced a decrease of OB-R expression on PCMDS surface after 24 hours (8.48±0.79% vs 27.16±2.11% in control conditions) (P<0.001)(Fig. 4e).

Using RT-PCR analysis, we established that α-linolenic acid (200 µM) decreased OB-Ra (p<0.05) and OB-Rb (p<0.05) mRNA expression after 24 hours (76.57±4.21 A.U and 54.14±27.52 vs 100 A.U. in control conditions) whereas others lipids analysed had no effect (Fig 4e).

### Leptin alone did not influence PCMDS but inhibited the effects of α-linolenic acid on PCMDS survival, Bcl-2 and OB-R expression

**Figure 5:**
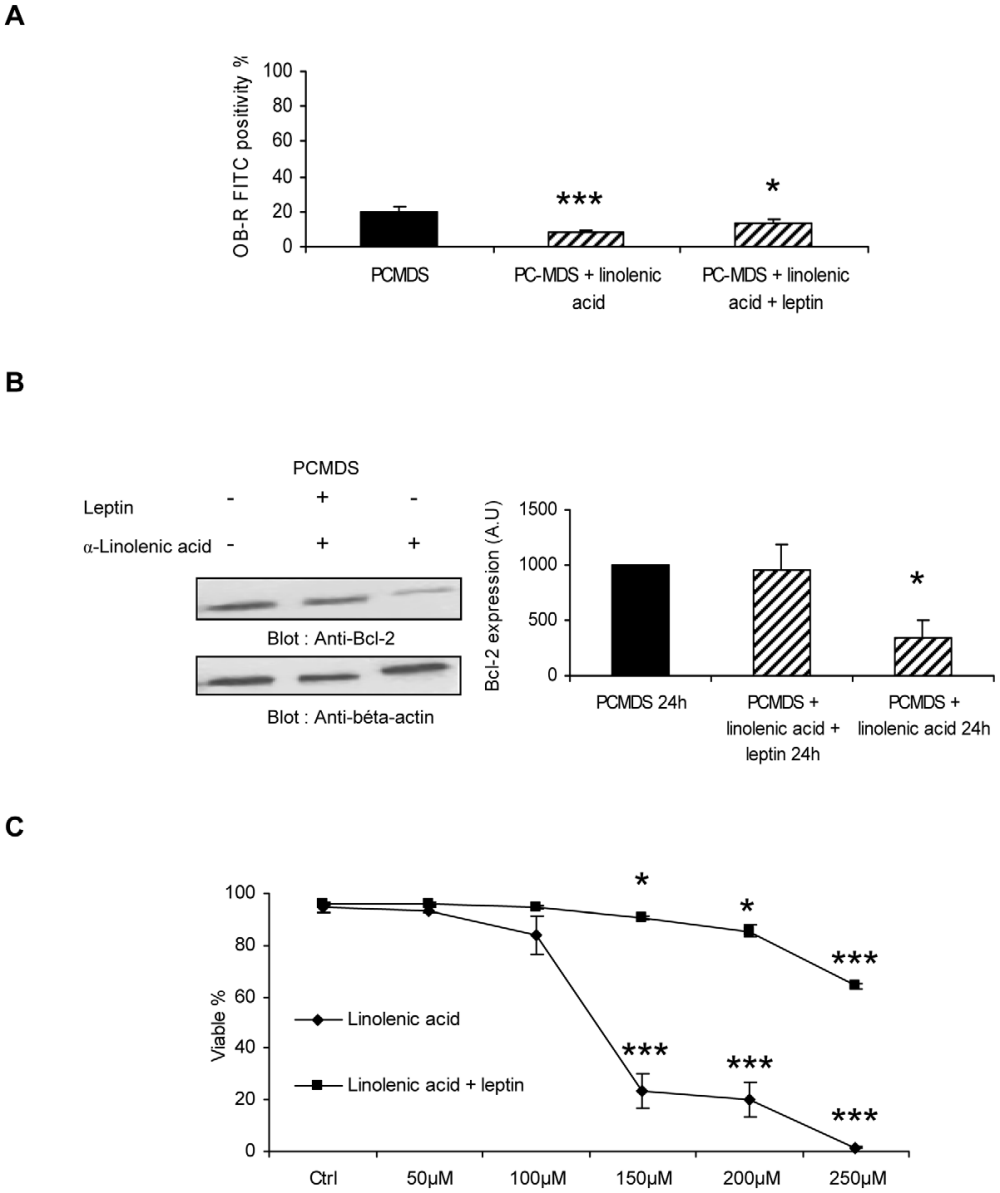
Leptin disturbed the effect of α-linolenic acids. **a** effect of 200 µM α-linolenic acids alone or with leptin (300pg/ml) on membranous OB-R expression in PCMDS after 24 hours (*black columns* PCMDS without stimulation; *hatched columns* PCMDS with stimulation). OB-R expression was analysed by flow cytometry. *P<0.05 and ***P<0.001 versus in control condition (Student *t* test)**. b** effect of 200 µM α-linolenic acids alone or with leptin (300pg/ml) on BCL-2 expression in PCMDS after 24 hours (*black columns* PCMDS without stimulation; *hatched columns* PCMDS with stimulation) measured by western blot. *P<0.05 versus in control condition (Student *t* test). **c** effet of α-linolenic acids with or without leptin (300 pg/ml) on PCMDS survival after 48 hours. *P<0.05 and ***P<0.001 versus in control condition (Student *t* test) *Columns*, mean of at least three independent determinations, *bars*. SD.

The high levels of leptin in co-cultures of FLFCs and PCMDS led us to analyse the effects of leptin on PCMDS cultured alone or in presence of fatty acids. Leptin alone (300 pg/ml) did not influence PCMDS proliferation, survival and OB-R expression. However, leptin inhibited partially the decrease in OB-R expression induced by α-linolenic acids (8.48±0.79% with α-linolenic acid vs 18.83±4.56% with α-linolenic acid and leptin) (p<0.01)(p<0.05) (Fig. 5a). Leptin also inhibited the decrease of BCL-2 expression induced by α-linolenic acids (956.54±231.21 vs 1000 A.U after 24 hours)(fig.5B), maintained PCMDS survival in the presence of low concentrations of α-linolenic acids (50 and 100 µM) and partially protected them from death induced by the higher doses (150 µM to 250 µM) (p<0.05) (150 µM : 90.4±0.55 vs 23.48±6.41% , 200 µM: 85.5±2.47 vs 20.2±6.83 and 250 µM : 64.26±0.90 vs 1.43±0.32)(Fig.5c). Leptin did not affected the decrease of proliferation induced by α-linolenic acids (data not shown).

### Low OB-R expression was associated with phospho-Akt inhibition and caspase activation

**Figure 6:**
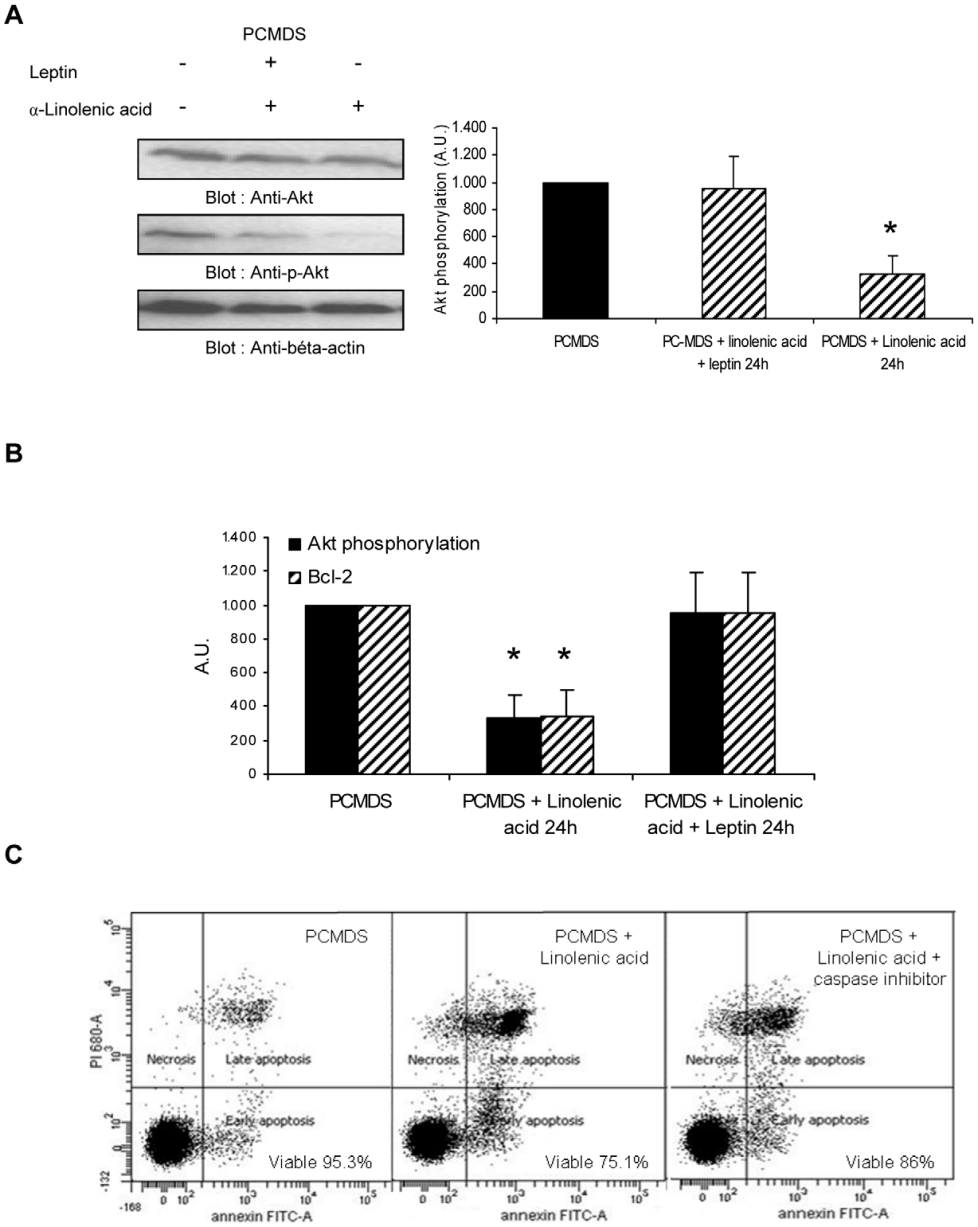
α-Linolenic acid inhibit AKT phosphorylation and induce caspase activation. **a** Akt phosphorylation in PCMDS stimulated by α-linolenic acid 24 hours with and without leptin (*black columns* PCMDS without stimulation; *hatched columns* PCMDS with stimulation) measured by western blot. *P<0.05 versus in control condition. **b** PhosphoAkt (*black columns*) and BCL-2 (*hatched columns*) level in PCMDS after α-linolenic acid contact with and without leptin. Akt phosphorylation and BCL-2 were measured by western blot. *P<0.05 versus in control condition (Student *t* test).*Columns*, mean of at least three independent determinations, *bars*. SD. **c** PCMDS survival in control, with α-linolenic acid or with α-linolenic acid and caspase inhibitor after 24 hours.

Phosphatidylinositol 3-kinase (PI3K) was the main signalling pathway of OB-R, implicated in the regulation of cell growth, proliferation and apoptosis. After 24 hours of contact with α-linolenic acids, a partial inhibition of Akt phosphorylation was observed (p<0.01)(333 ± 130 A.U vs 1000 A.U). Leptin addition restored the activation of PI3K/AKT pathway (Fig. 6a). The low level of phosphoAkt was associated with the low level of BCL-2 observed after 24 hours (Fig. 6b). As the antiapoptotic effect of AKT is principally due to partial inhibition of caspase activation [27]–[28], we used caspase inhibitor (R&D Systems Inc, France) and showed a significant inhibition (p<0,05) of PCMDS cell death induced by α-linolenic acid (85.26±1.66 vs 77.03±1.7%) (Fig. 6c).

## Discussion

Microenvironmental factors are suspected of influencing the evolution of chronic myeloid leukemias (CML). In this work we postulated that bone marrow adipocytes, leptin and lipids could be involved.

In the first step, we analyzed *in vitro* the interactions between adipocytes and two BCR-ABL positive cell lines using a T cell line (Jurkat) and a stromal cell line (HS-5) as controls. Adipocytes induced a decrease in BCR-ABL positive cell lines proliferation but did not influence Jurkat cells. By contrast, HS-5 stromal cells did not influence leukemic cells. This finding is in agreement with previous studies reporting that CML cells proliferate continuously even when being in contact with BM stroma [29]. In addition, it has been shown that morphologically identifiable adipocytes, almost absent in the bone marrow of leukemic patients [8], [30] reappear in high amounts during the remission period[13]. CML cell lines, inducing adipocytes but not HS-5 cell death *in vitro,* could be responsible for the disappearance of adipocytes observed *in vivo* [30].

In contact with adipocytes, leukemic cells of myeloid origin but not of lymphoid origin accumulate lipids. This lipid accumulation can be observed *in vivo* using Sudan Black B (SBB) staining [31]. Lipid accumulation in myeloid cells has previously been noted in some pathological conditions such as atherosclerosis [32] and asthma [33]. In cancer [18]–[19], this accumulation seems to protect cells from microenvironmental cytotoxins [34]–[35] and to induce inflammatory response [36]–[38] frequently observed in the BM of CML patients [39] and associated with reduced proliferation [34], [40]–[41].

The consequences of lipid accumulation appear to be dependent on the types of fatty acids accumulated. Our chromatographic analyses of the BM aspirates and biopsies of healthy patients showed a clear difference between the lipid composition of supernatant and cells of the BM microenvironment .The supernatant were rich in PUFA, while the cells were full of SFA and MUFA, fatty acids that are generally accumulated by tumor cells to protect themselves against external agents. An *in vitro* study has demonstrated that the presence of adipocytes protects leukemia cells from chemotherapeutic agents through Bcl-2 overexpression [42]. Our results showed that stearic acid increased the Bcl-2 expression, whereas others fatty acids (oleic and linoleic acids) had no effects. In contrast, α-linolenic acid decreased BCL-2 and OB-R expression in CML cell lines and reduced the proliferation and the survival of these cell lines. These observations are in direct agreement with two studies realised in HL-60 (Human promyelocytic leukemia cells) and U937-1 (Human monocytic cell line) cell lines where EPA (Eicosapentaenoic acid), a derivative of α-linolenic acid, once accumulated in the cell, reduced survival and proliferation [40]–[41]. Other studies focusing on breast cancer also showed an apoptotic and antiproliferative effects of omega-3 polyunsaturated fatty acids (PUFAs n-3) including α-linolenic acid [18], [43]. These findings are consistent with a study that showed that a cell with PUFAs n-3 was more sensitive to chemotherapy than the one filled with oleic and stearic acid [19].

This phenomenon seems to be specific to the myeloid lineage. Indeed, the majority of T-cell lines, previously characterized as Sudan Black B negative [31], do not accumulate fat. But some studies presented rare cases of SBB positive acute lymphoblastic leukemia (ALL) [44] as the Jurkat T cell line [45]. This rare occurrence might explain why small lipids droplets are observed in contact with PUFA. But unlike Cury-Boaventura *et al*, in our study we did not observe any effect of fatty acid on Jurkat cells proliferation and survival [46]. The reason of this discrepancy might be the presence of antioxydant in our culture medium.

Although the fish omega-3 fatty acids protective role against cancer is known, the role of α-linolenic acid, a PUFA n-3 found in high concentrations in vegetable oils, 5 to 10 times more prevalent [43] in the diet, remains controversial. α-Linolenic acid protects against atherosclerosis, a disease involving monocytes which accumulate fat and generate foam cells [47]. The effect of α-linolenic acid on increasing the risk of prostate cancer is still debated [47], [48].

According to different studies in tumor cells [49]–[50], the effects of α-linolenic acids in myeloid leukemia seemed to be directly related to PI3K pathway and Bcl-2 inhibition associated with caspase activation. Fatty acids act on normal or tumor cells via this pathway. PUFA n-3 inhibits, while PUFA n-6 and MUFA activate, the PI3K pathway [4], [18]. The PI3K pathway is activated in many cancers [3], [51]–[52] and is linked to the control of growth, migration, proliferation and apoptosis of tumor cells such as leukemic cells [3]–[4], [34]. This pathway is essential for the survival of leukemic cells [50] and is involved in the development of resistance to treatment with imatinib [22].

In our study, we have also shown that leptin production was increased when BCR-ABL positive cell lines were co-cultured with adipocytes. Leptin is known as a proliferative and antiapoptotic agent in many cancers [23], [51], [53]. According to recent studies, the effects of leptin involve its receptor (OB-R) and PI3K pathway [27], [54], the major signaling pathway of OB-R [21]. In our study, leptin alone had no effect on OB-R expression, proliferation or survival of CML. This might be explained by the difference in the amount of leptin used. Identical amounts to those observed in the co-culture medium were used, i.e. 300 pg / ml, whereas in others studies, the concentration of leptin used was about 30 to 3000 fold higher [53]–[54].

However, in the presence of leptin, the effects of α-linolenic acid on proliferation, survival, OB-R and BCl-2 were reduced. This phenomenon was also observed, after the addition of DHA (Docosahexaenoic acid), an other derivative of α-linolenic acid, in the presence and absence of leptin in rat glial and pituitary cells tumor [55]. Both our results and previous studies suggest that an adipocyte-rich composition of stearic and oleic acid promotes the development of leukemia, while α-linolenic acid exerts an inhibitory effect. Leptin can counteract α-linolenic acid inhibitory effects.

Therefore we suggest that at the onset of the disease, bone marrow adipocytes partially inhibited the expression of malignant leukemic clone. Cytokines produced by leukemic cells induce lipolysis of BM adipocytes. Then PUFA n-3 released from the adipocytes impairs CML proliferation and survival by inhibiting the PI3K pathway. Thereafter, these effects are soon inhibited by leptin released by adipocytes that also increased the lipolysis of adipocytes [56] releasing SFA and MUFA that protect CML from apoptosis by activating the PI3K pathway. A fat cell that loses its fat is destined to die [56]–[57]. This could explain why adipocytes are suffering in contact with CML and why adipocytes almost disappear in CML [30]. Then, in blast crisis, leukemic cells proliferate and survive without any influence by adipocytes, but under the influence of lipids supplied from blood LDL. After treatment, the reappearance of adipocytes could influence the survival of some residual malignant cells.
